# Which cardiac magnetic resonance parameters predict outcome in acutely reperfused non-ST-elevation myocardial infarction?

**DOI:** 10.1186/1532-429X-17-S1-P98

**Published:** 2015-02-03

**Authors:** Nils Dyckmanns, Dominik Buckert, Wolfgang Rottbauer, Peter Bernhardt

**Affiliations:** Internal Medicine II, University Ulm, Ulm, Germany

## Background

Clinical outcome after acute ST-elevation myocardial infarction (STEMI) has been shown to be predictable by myocardial salvage index (MSI) and microvascular obstruction as determined by cardiac magnetic resonance imaging (CMR). In the setting of non-ST-elevation myocardial infarction (NSTEMI) prognostic significance is less well investigated. Due to comparable incidence of major adverse cardiovascular events (MACE) in the setting of STEMI and NSTEMI the importance of risk stratification in NSTEMI patients is obvious. Aim of the present study was to evaluate CMR parameters with prognostic value for NSTEMI patients.

## Methods

In 60 patients presenting with NSTEMI, functional and volumetric analyses assessed by standard steady-state free-precession sequences, a T2-weighted black-blood fat-saturated spin-echo sequence for visualization of myocardial edema and late gadolinium enhancement imaging for quantification of infarct size was performed by CMR after acute coronary revascularization within 72 hours of symptom onset. For CMR measurements a 1.5 T whole-body scanner with a 32-channel phased-array surface coil was used. Major acute cardiovascular events defined as death and acute coronary syndrome were investigated for a follow-up period of three years after index event.

## Results

Median follow-up time was 34 ± 11 months after index event. In sum, 15 major adverse cardiovascular events occurred during the entire follow-up period. Mean CMR derived infarct size determined by LGE was 18.4 ± 13.5 % of LV mass, mean MSI was 34.2 ± 30.4 %. The mean area of microvascular obstruction was 1.0 ± 2.2 % of LV mass. When comparing survivals using the Log rank test, severely impaired left ventricular ejection fraction (LVEF ≤ 40%) was the only significant variable (HR 3.9; p = 0.02). Infarct size, microvascular obstruction and MSI did not differ significantly.

## Conclusions

Among patients presenting with NSTEMI there is substantial heterogeneity concerning the risk of major adverse cardiovascular events. In our cohort severely impaired left ventricular ejection fraction was the only predictor for adverse event yielding a hazard ratio of 3.9. To date prognostic significance of MSI and microvascular obstruction was mainly evaluated in STEMI patients but in terms of NSTEMI CMR parameters for prediction of MACE remain unsettled. Based on our results further studies are warranted to assess the prognostic value of CMR-derived parameters in the heterogenic group of NSTEMI patients.

## Funding

None.

